# Synergistic interaction of hTGF-β_3_ with hBMP-6 promotes articular cartilage formation in chitosan scaffolds with hADSCs: implications for regenerative medicine

**DOI:** 10.1186/s12896-020-00641-y

**Published:** 2020-08-27

**Authors:** Yijiang Huang, Daniel Seitz, Yan Chevalier, Peter E. Müller, Volkmar Jansson, Roland M. Klar

**Affiliations:** 1 Department of Orthopaedics, Physical Medicine and Rehabilitation, University Hospital of Munich, 81377 Munich, Germany; 2 BioMed Center Innovation gGmbh, 95448 Bayreuth, Germany

**Keywords:** Chitosan, Promotion, Adipose-derived stem cell, Articular Chondrogenesis, Bone formation, hTGF-β_3_, hBMP-6, Synergism, Validation

## Abstract

**Background:**

Human TGF-β_3_ has been used in many studies to induce genes coding for typical cartilage matrix components and accelerate chondrogenic differentiation, making it the standard constituent in most cultivation media used for the assessment of chondrogenesis associated with various stem cell types on carrier matrices. However, in vivo data suggests that TGF-β_3_ and its other isoforms also induce endochondral and intramembranous osteogenesis in non-primate species to other mammals. Based on previously demonstrated improved articular cartilage induction by a using hTGF-β_3_ and hBMP-6 together on hADSC cultures and the interaction of TGF- β with matrix in vivo, the present study investigates the interaction of a chitosan scaffold as polyanionic polysaccharide with both growth factors. The study analyzes the difference between chondrogenic differentiation that leads to stable hyaline cartilage and the endochondral ossification route that ends in hypertrophy by extending the usual panel of investigated gene expression and stringent employment of quantitative PCR.

**Results:**

By assessing the viability, proliferation, matrix formation and gene expression patterns it is shown that hTGF-β_3_ + hBMP-6 promotes improved hyaline articular cartilage formation in a chitosan scaffold in which *ACAN* with *Col2A1* and not *Col1A1* nor *Col10A1* where highly expressed both at a transcriptional and translational level. Inversely, hTGF-β_3_ alone tended towards endochondral bone formation showing according protein and gene expression patterns.

**Conclusion:**

These findings demonstrate that clinical therapies should consider using hTGF-β_3_ + hBMP-6 in articular cartilage regeneration therapies as the synergistic interaction of these morphogens seems to ensure and maintain proper hyaline articular cartilage matrix formation counteracting degeneration to fibrous tissue or ossification. These effects are produced by interaction of the growth factors with the polysaccharide matrix.

## Background

Healthy articular cartilage lacks self-repairing capacities due to its avascular structure. This makes self-regeneration and self-healing impossible, unlike bone, which can lead to functional limitations and pain eventually associated to osteoarthritis [1–4]. Compared to other therapies, autologous chondrocyte implantation has emerged as a promising technique in orthopedic surgery to treat cartilage defects [5–9]. However, autologous chondrocyte implantation suffers from several limitations. Often a cartilage graft is harvested from an articular joint, which can cause distinct donor site morbidity, limiting the supply of autologous chondrocytes [10–12]. Stem cell-based therapies are an alternative to overcome the poor self-repair capacity of cartilage, where, under the principle of tissue engineering, an insoluble substratum is combined with soluble signals [13–15]. For cartilage regeneration, chondrocytes or stem cells are often combined with a biomimetic biomaterial that supports the formation of neo-cartilage tissue with the typical characteristics of hyaline articular cartilage [16].

Extracellular matrix-like scaffolds can provide a structural template for cartilage development and also serve as a substrate that helps to facilitate cell attachment, proliferation, differentiation to the desired phenotype and integration into the adjacent cartilage [17–19]. The type of biomaterial and the architecture on cellular scale are key elements in the development of new materials for causing targeted stem cell differentiation into specific tissue types [20]. With a wide range of natural and synthetic polymers available, selecting the appropriate biomaterial is crucial [21, 22], as the material needs to both provide the necessary clues for cell development and differentiation and possess excellent biocompatibility for safe implantation. The polysaccharide chitosan, a component of crustacean and insect exoskeletons [23–25], is a copolymer of glucosamine and N-acetyl-glucosamine, obtained by the deacetylation of chitin. It is the most widely used biopolymer in various biomedical applications because of its potential to stimulate hemostasis and accelerate the regeneration of damaged or lost tissues in the process of wound healing [26, 27]. It has a hydrophilic surface that enhances cell adhesion, proliferation and differentiation, efficiently attracting fluids and cells to the defect site [28]. Chitosan has been shown to mimic native matrix components in their interaction with developing chondrocytes and possess excellent biocompatibility [29], biodegradability and physicochemical properties mimicking native chondrogenic matrix, making it suitable for cartilage tissue engineering [27, 30]. When combined with predisposed stem cells and morphogens it could become a viable alternative to other cartilage tissue engineering candidates. However, as our previous research has shown, chitosan on its own has only very limited articular cartilage forming capabilities which has to a great extent limited its use clinically [31].

In the chondrogenic differentiation process of mesenchymal stem or stromal cells (MSCs), members of the transforming growth factor β (TGF-β) supergene family play a crucial role [32–34]. Building on previous results for the interaction of TGF-β with chitosan, we aim to investigate whether combinations with other TGF-β superfamily members, specifically hBMP-6, increases articular cartilage formation potential as compared to the effect of TGF-β_3_ used alone or in combination with IGF-I. TGF-β_3_ has been described in various studies to promote cartilage repair and accelerate cartilage differentiation, upregulating the expression of genes typical for the formation of cartilage in hADSCs [33, 35]. The same genes, however, are activated in growth plate chondrocytes when endochondral ossification ensues. A previous study documented that TGF-β_1_ and TGF-β_3_ have a similar effect on cell proliferation, gene expression and articular cartilage biosynthetic activity in ADSCs cultured in alginate beads [36]. Cals et al. [37] reported that no significant differences in total collagen and GAG formation could be observed among MSCs cultured in medium containing the three TGF-β isoforms, respectively. Some other previous studies found that TGF-β_3_ was more efficient and potent than TGF-β_1_ in enhancing hADSCs and MSCs chondrogenic differentiation [38]. Other studies again have also shown that TGF-β_3_ is very beneficial for cartilage as it stimulates chondrocytes in vitro by inducing the elevation of proteoglycans and the production of collagen type II [32, 34, 39]. However, studies in a primate model have consistently shown that hTGF-β_3_ induces endochondral ossification rather than true articular cartilage formation in vivo, with recent research suggesting that the addition of human bone morphogenetic protein 6 (hBMP-6) promotes formation of hyaline, articular-like cartilage formation of both MSCs and hADSCs [31, 40–43].

Cellular chondrogenic differentiation pathways are influenced by BMPs via specific type I and type II BMP-receptors, activating Smads pathways [44]. BMP-6 is mainly expressed in cartilaginous tissue, in which mesenchymal cell differentiation into chondrocytes is stimulated, promoting the synthesis of chondrocytes and articular cartilage-specific glycoproteins [45]. At the same time, BMP-6 is also a known factor involved in the induction of bone formation [46], indicating a multi-functionality in the regulation of bone and cartilage cell development similar to most BMPs and other growth factors of the TGF-β superfamily. Though diverse groups have shown that BMP-6 can stimulate chondrocyte and cartilage formation [47, 48], to date results remain unspecific as to whether hyaline articular or endochondral ossification type cartilage is formed, making interpretations or validations problematic.

As various studies have assessed the biocompatibility and tissue engineering capabilities of pure and porous chitosan scaffolds with ADSCs, the primary aim of the present study was determining if the in vitro cartilage formation potential can be directed towards lasting, hyaline articular tissue when using a chondrogenic medium supplemented with hTGF-β_3_ and hBMP-6 as opposed to the standard chondrogenic medium normally containing only hTGF-β_3_. Subsequently, the study also sought to validate our previous research [31] that the matrix formation was articular and that hTGF-β_3_ alone causes endochondral bone formation and does not support articular chondrogenesis. Answering these questions can not only lead to improved in vitro models for consistent hyaline cartilage formation with biomimetic biomaterials, but also provide valuable information for clinical therapeutic articular cartilage repair, possibly preventing long-term degeneration of the treated site.

## Results

### Distribution and growth of hADSC in porous chitosan-GA scaffolds

**Fig. 1:**
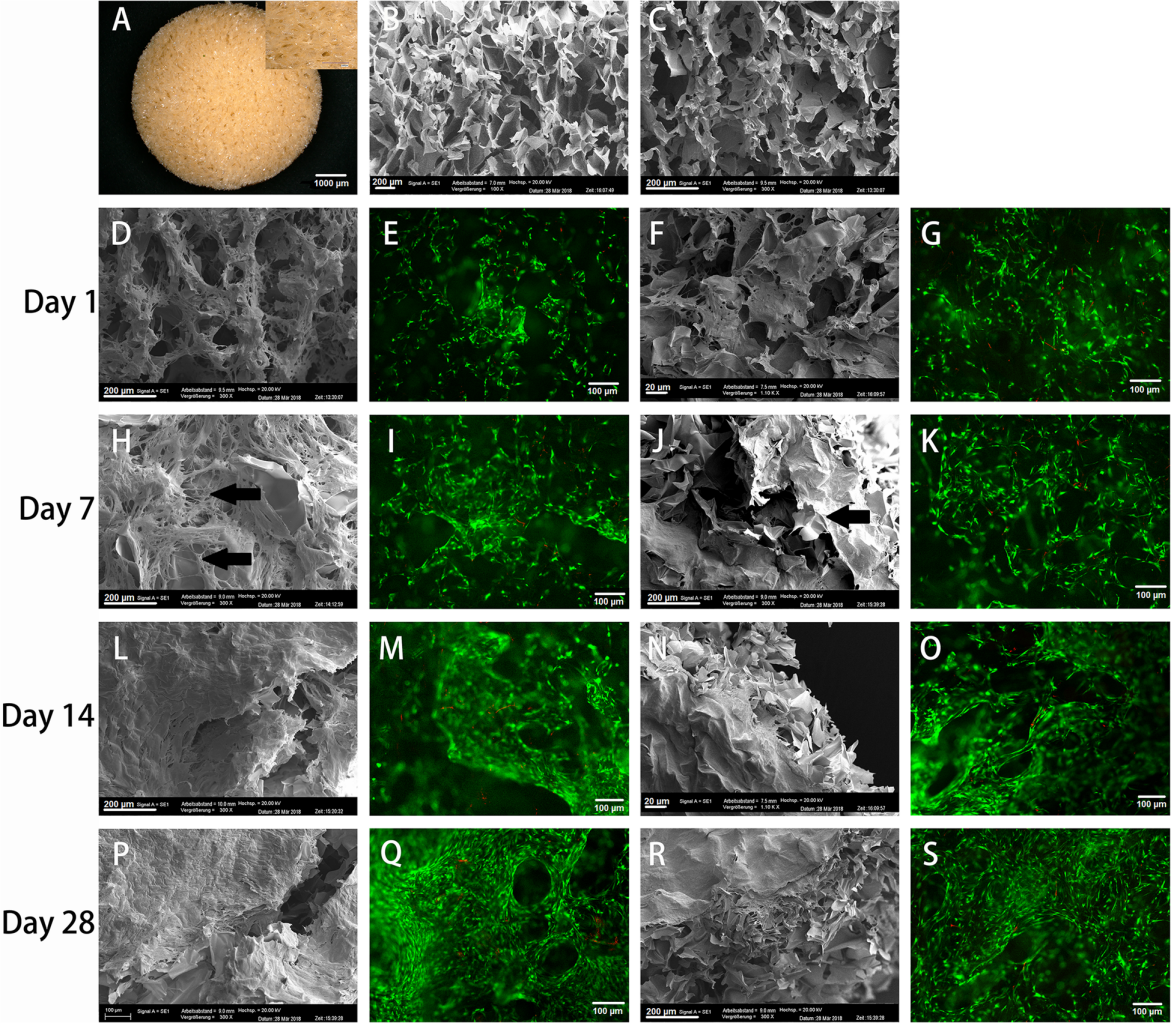
Scaffold characteristics and cell distribution in culture with hADSCs at different time points. **a** Stereomicroscopic image of the dry scaffold, showing sponge-like uniform porous structure. **b** Scanning electron microscopy images of chitosan scaffolds before and (**c**) after incubation with medium (cell-free blank). Image in **B** was used for pore size determination. Images **d-s** demonstrate cellular growth on days 1 (**d-g**), 7 (**h-k**), 14 (**l-o**) and 28 (**p-s**), the first two images showing growth with hTGF-β_3_ only, the second with hTGF-β_3_ + hBMP-6. Grey-scale images (first and third column) are SEM images, fluorescent images (second and fourth column) are live/dead stain with green = Calcein AM for live and red = Ethidium bromide for dead cells. Collagenous fibrous matrix (**black arrows**) (**h-k**) For (**a**) magnification was set at 4x; SEM magnifications were set at 100x (**b**), 300x (**c, d, h, j, l, p, r**), 1.10Kx (**f, n**); Live/Dead magnifications were set at 10x (**e**, **g**, **i**, **k**, **m**, **o**, **q**, **s**)

The chitosan scaffolds used in this study appeared as a soft and highly porous sponge-like cylinders (Fig. 1a). Pores were fairly uniform and showed irregular morphology (Fig. 1b), with porosity determined as 211 ± 66 μm from image analysis (length of cross-section measurements using ImageJ, NIH). The pore size distribution of the scaffolds used was overall evenly distributed and isotropic as a result of an optimized freeze-drying process (43). Mechanical properties could not be measured directly, as compression forces were below the range of our equipment, but they can be considered similar as described in other publications, as reviewed e.g. by Levengood et al. 2014 [49]. Values between 0.3 MPa tensile strength and 15.7 kPa [50] are given. After lyophilization, and especially with glutaraldehyde-crosslinking, the material does not swell in medium and retains sufficient rigidity to transfer mechanical cues to the cells.

**Fig. 2:**
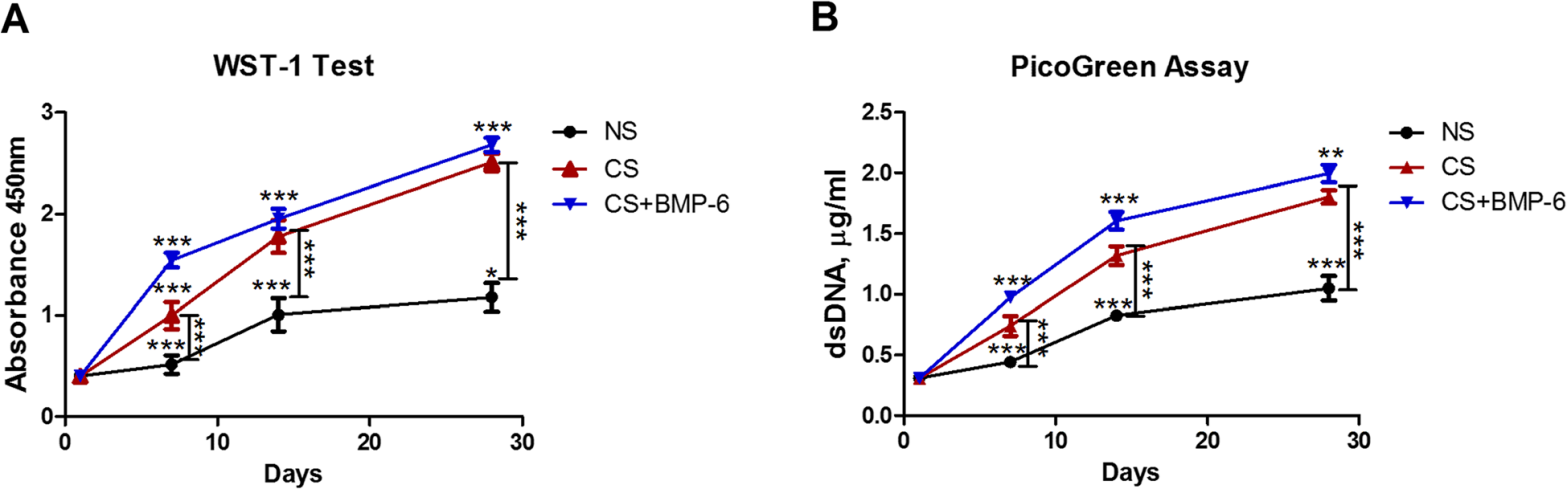
**a** WST-1 (cell viability) and **b** PicoGreen (cell proliferation) assays for hADSCs on chitosan scaffolds cultured with normal (NS), standard chondrogenic (CS) or modified chondrogenic + hBMP-6 (CS + hBMP-6) medium. (***p < 0.001)

**Fig. 3:**
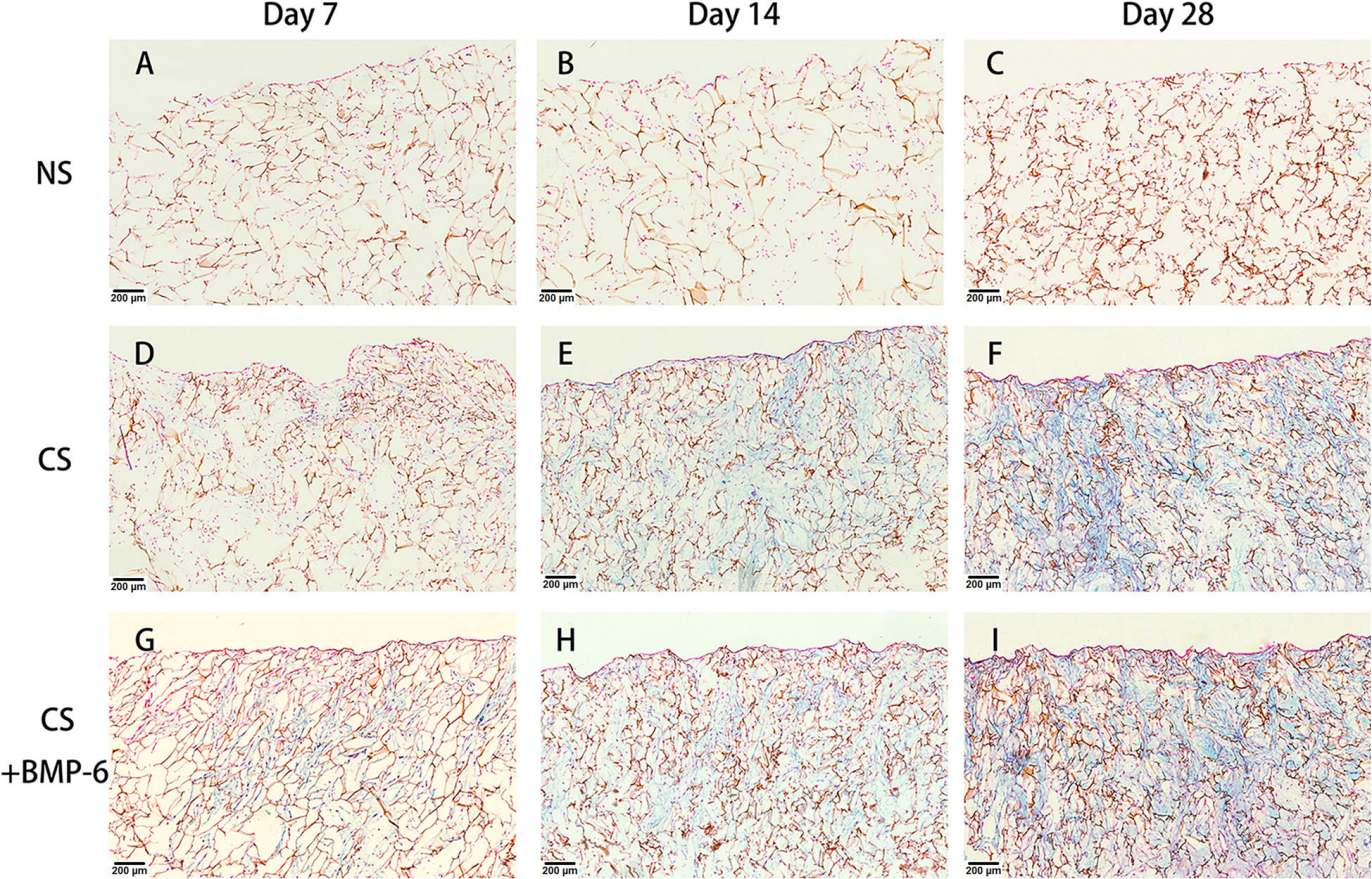
Alcian blue staining for GAG in chitosan scaffolds seeded with hADSCs cultured in either normal (NS) (**a-c**), standard chondrogenic (CS) (**d-f**) or modified chondrogenic + hBMP-6 medium (CS + hBMP-6) (**g-i**) after 7, 14 and 28 days. Magnification 40x (Bar scales: 200 μm)

Cells appeared well attached after 24 h (Fig. 1d-g) and distributed throughout the scaffold. Despite static culture, hADSC continued to grow well in the entire construct, forming abundant matrix (Fig. 1h-k). Matrix formation was strong with both hTGF-β_3_ and hTGF-β_3_ + hBMP-6, with no obvious difference between both treatments. In comparison, cells cultured without growth-factors showed less proliferation and matrix development (Figs. 1, 2 and 3). By day 14, abundant fibrous matrix had already formed (Fig. 1l-o) and after 28 days, the scaffold structure had completely disappeared under the newly formed matrix (Fig. 1p-s). The development and the stimulating effect of the growth-factors did not seem impaired in the inner pores as compared to the outer rim, as no differences were found in SEM analysis.

In order to evaluate cell viability and proliferation on the hADSCs-seeded scaffolds, a WST-1 test in combination with a PicoGreen assay was performed 24 h after cell seeding and subsequently after 7, 14 and 28 days of incubation. Both cell viability and cell number, as indicated by DNA amount, increased progressively over the 28 day incubation period, indicating a steady increase of cells in all experimental groups, with a characteristic decrease in slope after 14 days marking the onset of differentiation (Fig. 2). The course of both parameters was alike and there was no difference between CS cultures and those treated with additional BMP-6. From day 7, there was a clear gap between cultures with growth-factor medium and those with proliferation medium, which widened in the further course (Fig. 2). When differentiation starts, cells usually switch from multiplication to matrix production, so growth factors often inhibit proliferation as compared to normal media. In this case, however, both cell proliferation (Fig. 2b) and cellular activity (Fig. 2a) were significantly higher under the influence of differentiation medium.

### Histological and immunofluorescent analyses of matrix formation

**Fig. 4:**
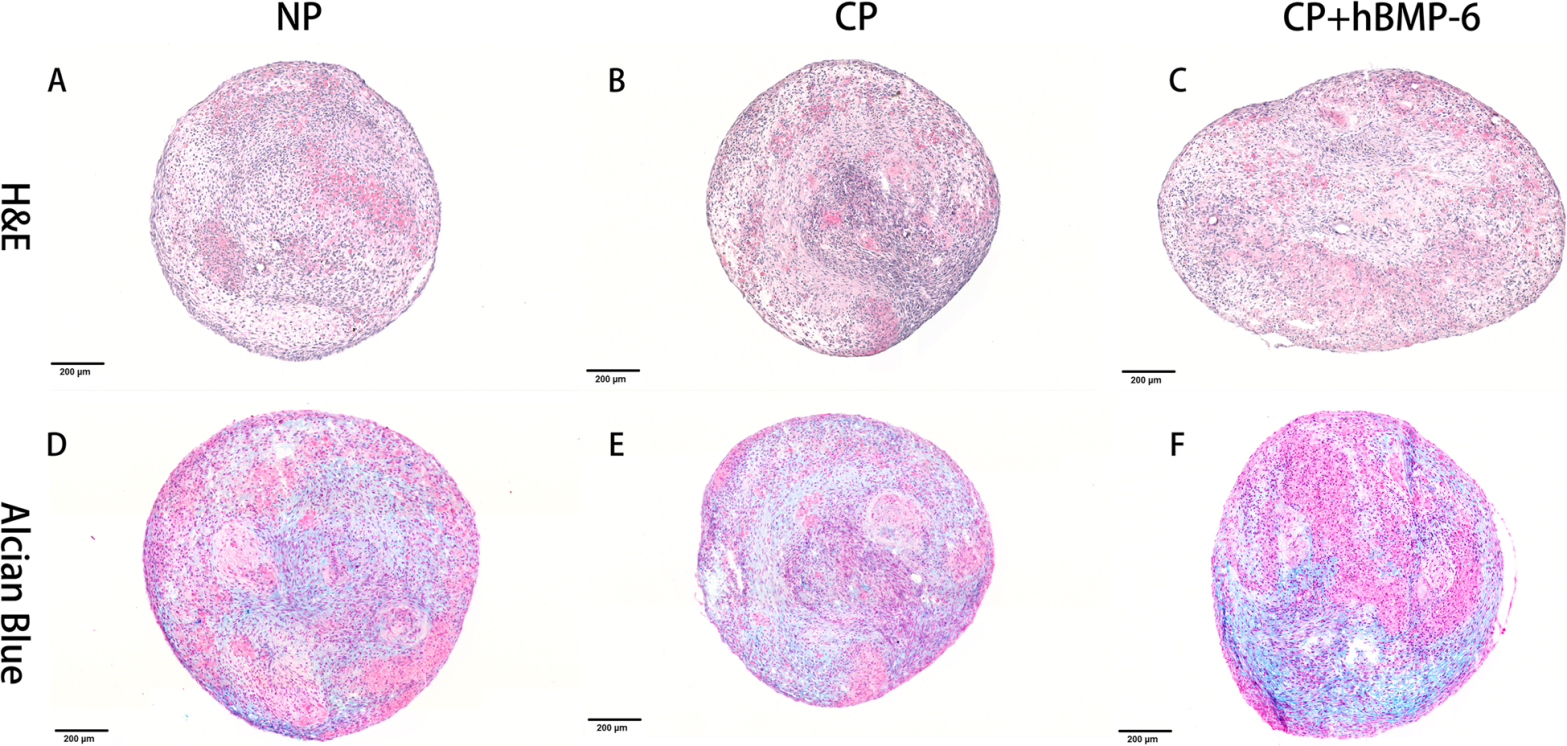
H&E and Alcian blue staining of 3D hADSCs pellets cultured in either normal (**a**, **d**), standard chondrogenic (**b**, **e**) and modified chondrogenic + hBMP-6 medium (**c**, **f**) at day 28. H&E staining of the 3D hADSCs pellet cultures in normal medium (NP). Magnification 20x (Bar scales: 200 μm)

**Fig. 5:**
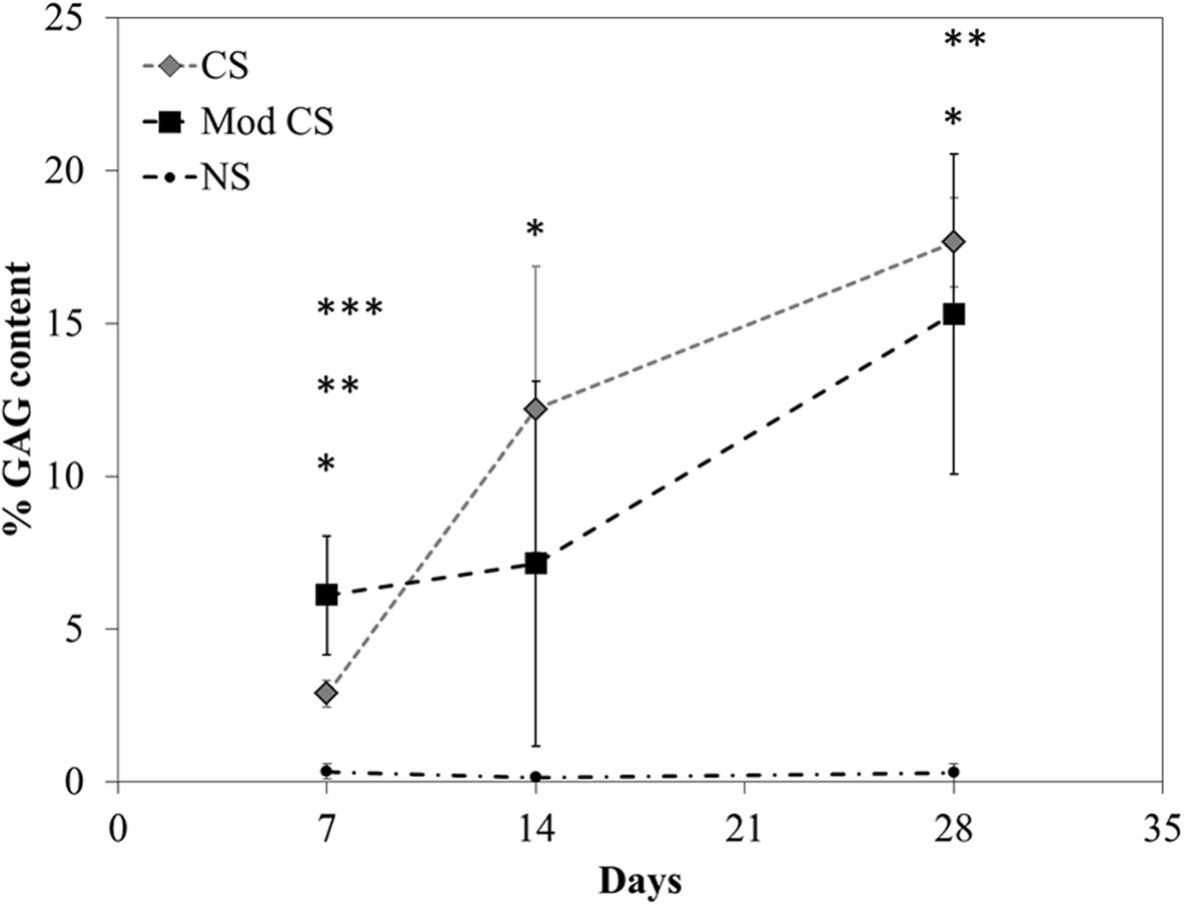
Histomorphometrical assessment of GAG (alcian blue) staining percentage between NS, CS and CS + hBMP-6 groups. (*p < 0.05, **p < 0.01, ***p < 0.001)

The quality of the matrix formed was analyzed using Alcian blue staining for cartilage GAG in both scaffold (Fig. 3) and pellet cultures (Fig. 4). A clear enhancement of Alcian blue positive matrix formation by the application of growth factors was observed in the scaffolds (Fig. 3) supported by the histomorphometrical assessment (Fig. 5). The matrix was distributed throughout the scaffold with both treatments, with regional differences in density that are commonly observed after in vitro culture without mechanical stimulation at this stage.

A different picture arose for cell pellets. Pellet culture mimics cartilage nodule formation, giving a natural environment for chondrocyte differentiation, thus reliably leading to some form of cartilage formation in most cases. Accordingly, a certain amount of Alcian-positive matrix was found after culture in normal medium (Fig. 4d). Pellets cultured with hTGF-β_3_ reach the same size, but had a much higher cellular density, coalescing with a heterogeneous histological picture (Fig. 4b), while the combination with hBMP-6 lead to much larger pellets with more matrix surrounding the cells (Fig. 4c). Although the pellets were not immunostained, the Alcian blue results (Fig. 4e, f) can be interpreted such that with hTGF-β_3_ only, a very heterogeneous, partly condensed, partly hypertrophic tissue was formed, while the mature parts of pellets cultivated with hBMP-6 show strong and evenly distributed positive staining for cartilage matrix GAG.

**Fig. 6:**
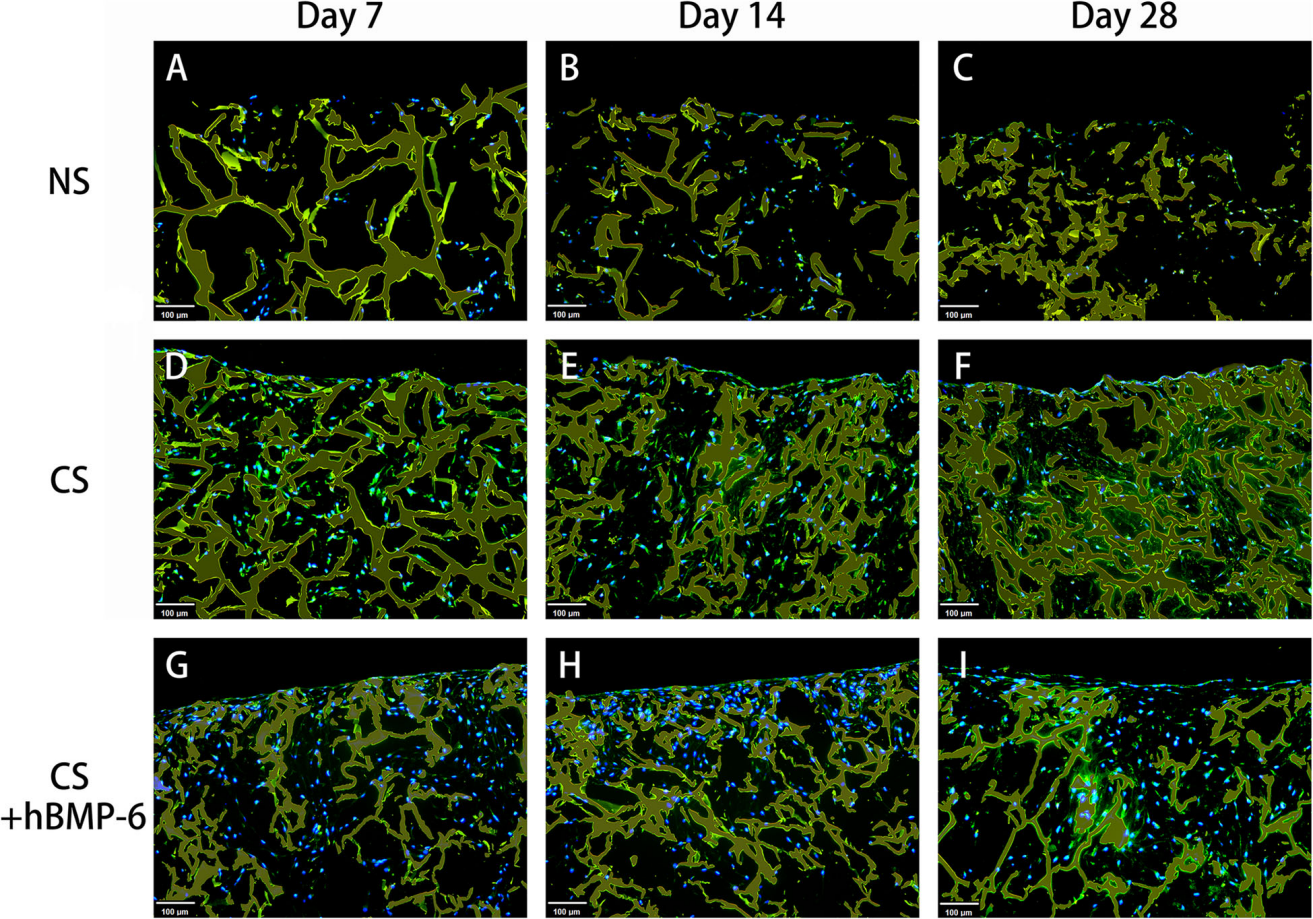
Immunofluorescence staining of aggrecan (**green**) at day 7, 14 and 28 in chitosan scaffolds with hADSCs cultured in normal (NS), standard chondrogenic (CS) or modified chondrogenic + hBMP-6 medium (CS + hBMP-6). The chitosan scaffolds fluoresced yellow, whereas living cell nuclei fluoresced **blue**. Magnification set a 10x

**Fig. 7:**
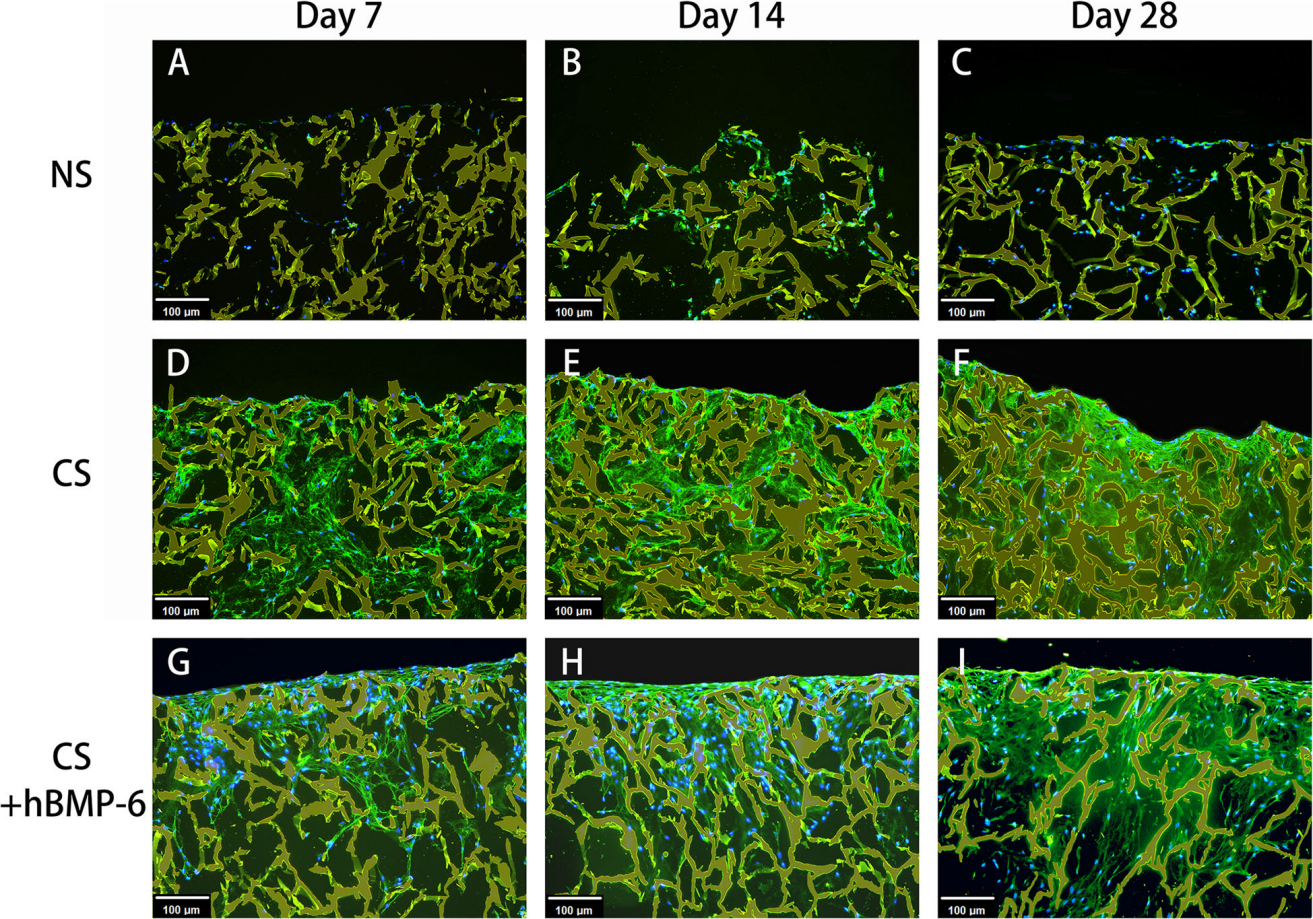
Immunofluorescence staining of collagen type II (**green**) at day 7, 14 and 28 in chitosan scaffolds with hADSCs cultured in normal (NS), standard chondrogenic (CS) or modified chondrogenic + hBMP-6 medium (CS + hBMP-6) . The chitosan scaffolds fluoresced yellow, whereas living cell nuclei fluoresced **blue**. Magnification set a 10x

**Fig. 8:**
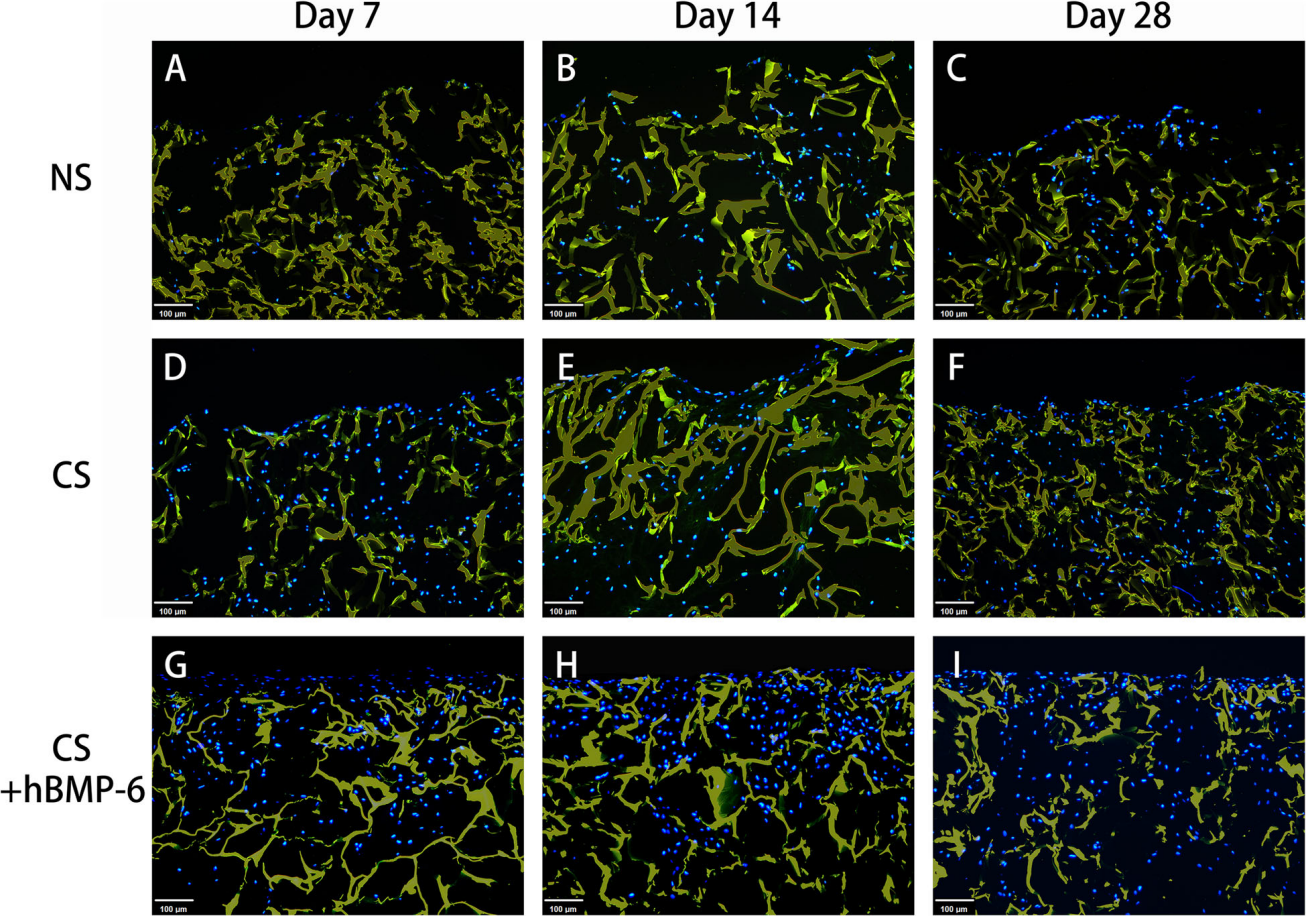
Immunofluorescence staining of collagen type I (**green**) at day 7, 14 and 28 in chitosan scaffolds with hADSCs cultured in normal (NS), standard chondrogenic (CS) or modified chondrogenic + hBMP-6 medium (CS + hBMP-6). The chitosan scaffolds fluoresced yellow, whereas living cell nuclei fluoresced **blue**. Magnification set a 10x

Using immunofluorescence for a more detailed analysis of the matrix formed, the deposition of aggrecan (ACAN), an important and specific component of cartilage matrix, appeared to be slightly higher with hTGF-β_3_ treatment only (Fig. 6). No ACAN was expressed without growth factors. The quality of tissue engineered cartilage is strongly defined by the relation of collagen II to collagen I formation. Collagen I does not belong to functional hyaline cartilage, with collagen II fibers giving the basic framework of the cartilage matrix. Here, almost no collagen II was found when no growth factors were applied (Fig. 7), but also no collagen I (Fig. 8). Because proliferating chondrocytes usually form some amount of collagen I matrix and to exclude antibody functionality, immunohistochemical staining was performed (Suppl. Fig. 1) to validate collagen I results. A weak signal was detected in both normal proliferation medium and hTGF-β_3_ at day 14 and 28 (Suppl. Fig. 1B, C, E, F) yet remained absent throughout hTGF-β_3_ + hBMP-6 groups. It is therefore probable that the background noise form scaffold absorbing the immunofluorescent signal is affecting the detection of the collagen I and not the antibody itself. Abundant collagen II could be found with hTGF-β_3_ treatment, whereas the addition of hBMP-6 seemed to reduce the amount of this protein by day 28 (Fig. 7). Collagen X (Suppl. Fig. 2) was not detected in control and treatment groups.

The differences between chitosan-scaffold and the pellet culture were striking. Embedded in the polysaccharide, hADSC respond to both growth-factor regimes with abundant matrix formation, with no differences in distribution and amount of Alcian-blue positive matrix and even a stronger formation of collagen II under hTGF-β_3_ only. In pellet culture, the hADSC remain limited in matrix formation without additional hBMP-6, forming a heterogeneous tissue with apparently hypertrophic regions. The immunofluorescent matrix analysis has given unclear results in this case. In order to monitor more clearly the differences gene expression was employed.

### qRT-PCR of in vitro chondrogenic differentiation

**Fig. 9:**
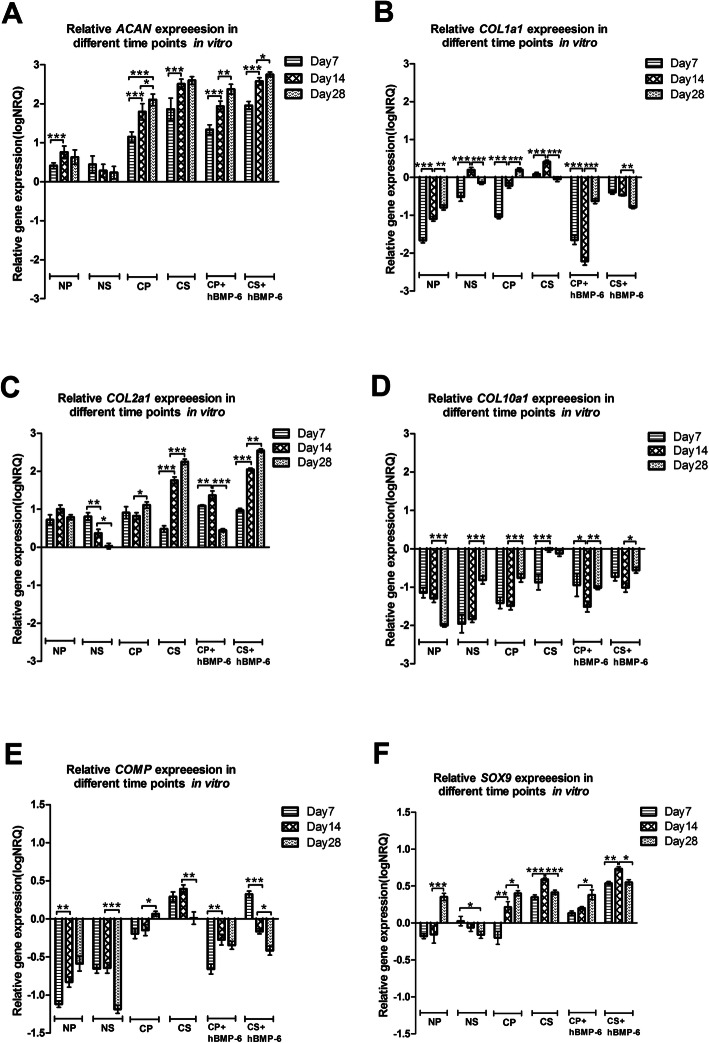
Relative gene expression quantity of (**a**) *ACAN*, (**b**) *COL1A1*, (**c**) *COL2A1*, (**d**) *COL10A1*, (**e**) *COMP* and (**f**) *SOX9* between all culture groups (N = normal medium; C = chondrogenic medium, *P* = 3D Pellet; S = chitosan scaffolds,). (*p < 0.05, **p < 0.01, ***p < 0.001). The baseline 0 represents untreated hADSCs in monolayer, which was the normalisation factor

To evaluate chondrogenic gene expression between the NS, CS, CS + hBMP-6, NP, CP and CP + hBMP-6 groups, relative qRT-PCR gene analysis was performed on in vitro samples, monitoring the relative change in transcription of *ACAN*, *COL1A1*, *COL2A1*, *COL10A1*, *SOX9* and *COMP*. The results represent a snapshot of the above genes at day 7, 14 and 28 after culturing with standard chondrogenic, modified chondrogenic induction medium or normal medium in chitosan-based scaffolds seeded with hADSCs or in the form of a 3D pellet. The results have been normalized to four reference genes (*ACTB*, *RPLP0*, *TBP*, *POLR2e*), expressed as log_10_CNRQ (calibrated normalized relative quantities, CNRQ). Relative expression of every gene in different groups but at the same time point is shown in Suppl. Fig. 3, whereas of every gene at different time points but in the same group is shown in Fig. 9.

The hyaline cartilage matrix *ACAN* and *COL2A1* expressions were found to be up-regulated in all groups (Fig. 9a, c) and increased significantly by day 28 in both pellets and cell-scaffold constructs treated with either chondrogenic medium (*p* < 0.05) (Fig. 9a, c). *ACAN* was much higher in all growth factor- (GF-) treated groups and collagen II in both GF-treated scaffold cultures, and both increased in expression with culture time (Fig. 9a, c). Interestingly, for chitosan-scaffold cultures, both genes showed a greater increase in expression under either GF-treatment, decreasing in normal medium, whilst in pellet culture, *ACAN* slightly increased and *COL2A1* remained constant over time in normal medium, with no significant increase of the latter under hTGF-β_3_ treatment. With additional hBMP-6, pellets expressed slightly more *COL2A1* on day 14, but significantly less on day 28.

The cartilage-oligomeric matrix protein (COMP) is an important regulator of matrix formation; the nuclear transcription factor SOX9 is considered the central orchestrating signaling molecule of cartilage differentiation. Both clearly responded to growth-factor treatment, more clearly so for scaffold cultures. *COMP* and *SOX9* were both upregulated in CS group at all-time points and the expression increased significantly by day 14, greatly exceeding values for pellets, but decreased slightly at day 28 (Fig. 9e, f), whereas in the CS + hBMP-6 groups, *COMP* was upregulated briefly at day 7 after which it significantly decreased by day 28 (Fig. 9e, f). Both genes were more strongly expressed with time in pellet cultures, *COMP* remaining downregulated in proliferative and becoming slightly upregulated in hTGF-β_3_ medium, yet further downregulated with hBMP-6 (Fig. 9e, f). *SOX9* expression in pellets with normal medium was downregulated at day 7 and 14, but upregulated at day 28, whereas being downregulated at all-time points in normal medium with scaffolds group (Fig. 9e, f). As in scaffolds, *COMP* expression in pellets was significantly higher in chondrogenic groups at all-time points, while contrary to the former, SOX9 was not consistently so, expression levels being more or less equal for all treatments in pellets on day 28 (Suppl. Fig. 3E, F).

When analyzing cartilage matrix formation, it is insufficient to look at existing expression of positive markers, as these are common also in matrix destined for hypertrophy and mineralization. Here, *COL1A1* and *COL10A1* were included as negative markers for differentiation towards articular cartilage, as they are indicators for endochondral bone formation [54, 55]. The qRT-PCR results for those genes showed some similarities with the optical impression of histological images (Suppl. Figs. 1, 2).

For the fibrous tissue component *COL1A1* was generally downregulated, yet slowly increasing in pellet cultures, higher in scaffolds with normal medium but clearly upregulated in scaffolds and pellets with hTGF-β_3_ treatment (Suppl. Fig. 3B). Similar to dedifferentiation processes in 2D culture, it increased with time in all treatments except for scaffolds supplemented with hTGF-β_3_ + hBMP-6, where it was clearly and progressively downregulated (Fig. 9b). In pellet culture, downregulation of *COL1A1* was even stronger with hBMP-6, reaching the lowest level of all samples.

*COL10A1* expression was downregulated in both pellet cultures and scaffolds cultures with normal medium (Suppl. Fig. 3D), but intriguingly decreasing in expression in NP only, increasing towards day 28 in the other two (Fig. 9d). Similar to collagen I, this clear signal of beginning hypertrophy was increased in pellets by hTGF-β_3_ treatment in hADSC. Most striking in the expression pattern of *COL10A1* however is the difference between pure hTGF-β_3_ treatment and the addition of hBMP-6 in scaffold cultures. The expression level rose to the point of upregulation with hTGF-β_3_, but remained low with additional hBMP-6. There was no such contrasting influence on chondrocytes in pellet culture.

In interaction with the chitosan matrix, hTGF-β_3_ had a stronger effect on chondrogenic gene expression in hADSC, but also induced hypertrophy, part of which was rescued by adding hBMP-6. Although histological analysis revealed stronger differences between growth factors samples with and without additional hBMP-6 for pellets and not for scaffolds, qRT-PCR showed an effect of hTGF-β_3_ on the expression of *COL10A1* in scaffolds that was not found in pellet cultures. Moreover, the influence of hTGF-β_3_ medium on the expression of *COL2A1*, *COL1A1*, *COMP* and *SOX9* was much stronger in scaffolds, while the “rescuing” effect of additional hBMP-6 was much weaker for *COL1A1* and *COMP*.

## Discussion

Articular cartilage regeneration remains a challenging clinical task [2, 4, 51], despite several promising approaches using adapted scaffold materials and a combination of stem cells with growth factors [52–56]. Biomaterials used in cartilage tissue engineering need to be highly biocompatible, biodegradable, as well as possessing the adequate biomechanical properties and geometric organization that further support cell attachment, proliferation and differentiation [17–19]. As such, the ideal scaffold should mimic the extracellular cartilage matrix, in both form and function, and retain the phenotype of differentiated stem cells [57]. One such material is chitosan, a polysaccharide derived from the exoskeleton of arthropods, which has shown to be one of the more advantageous substances that has been widely investigated and used in various derivative forms in tissue engineering as well as for clinical applications [58]. In particular, scaffolds composed of poly(lactic-co-glycolic acid) (PLGA)/chitosan have been shown to support chondrocyte development from hADSCs and to form articular cartilage in a lagomorph model over 12 weeks [20, 59, 60]. Compared to chondrocytes isolated from mature cartilage and BM-MSCs isolated from bone marrow, hADSCs can be harvested in large amounts and are readily accessible [61]. Human ADSCs can be expanded in stable cultures of undifferentiated cells and can easily be differentiated into chondrocyte-like cells in vitro under specific culture conditions, maintaining the chondrogenic phenotype even in vivo after transplantation [62, 63]. However, as shown in our previous study [31], the use of hADSCs cultured on a pure, elastic and porous scaffold composed of glutaraldehyde-cross-linked chitosan is inadequate for articular cartilage regeneration. Without the correct soluble signals, the biomimetic matrices can only assist new tissue formation relying on additional morphogens to facilitate faster tissue growth in the regeneration for large defects. Yet, the correct signaling proteins needed to for the formation of stable hyaline cartilage (reviewed in [64]) within biomaterials have not been properly identified. Cartilage regeneration approaches using stem cells generate chondrocytes that, whilst depositing a chondrogenic-like matrix, have a strong tendency to either deposit large amounts of collagen I in a fibrous tissue or, even more often, go into hypertrophy in a mineralizing matrix that initiates endochondral ossification [65]. When following up on attempts to illuminate underlying mechanisms and improve the clinical situation, methodological inconsistencies appear, producing non-standardized and non-reproducible results. Indeed, some morphogens such as the hTGF-β_3_, accepted as a common chondrogenic stem cell differentiation morphogen [66], have been shown to have undergone evolutionary and functional variation, especially in regard to induction of bone formation between different animal models [67, 68]. It is therefore not clear if hTGF-β_3_ can truly form “articular cartilage” on biomaterial carriers or if a synergy with at least one more morphogen, such as used in previous studies with hBMP-6, is required [31, 43] to achieve this. This question was therefore assessed in the present study in which we sought to validate our previous results that hADSCs cultured in chitosan only form hyaline matrix in the presence of hTGF-β_3_ + hBMP-6, proving that a proper morphogen combination in the right carrier matrix is necessary to form the correct tissue type. It was further the intention to show that hTGF-β_3_ alone does not support hyaline articular formation but leads to hypertrophy that finally ends in bone formation, as suggested in studies by Ripamonti et al. [40–42] and Klar et al. [69].

The in vitro viability, proliferation and differentiation capacity of hADSCs cultivated in lyophilized scaffolds of glutaraldehyde-cross-linked chitosan, as previously observed, increased stably with culture time over the 28 days. The scaffolds with their porous structure enabled viability, migration and proliferation of hADSCs. Results from the Live/Dead assay demonstrated that the hADSCs exhibited very good adhesion and biocompatibility on the biomaterial. Comparing the results from this assay from 14 days onward between chondrogenic and control groups, a strongly enhanced cell proliferation in chitosan scaffolds with chondrogenic differentiation medium demonstrated a positive interaction of the carrier with the morphogens. Scanning electron microscopy clearly showed cells colonizing the scaffolds, forming a lush fibrous matrix with denser layers occurring on the periphery of the devise by 28 days. This effect has to be attributed to the influence of hTGF-β_3_, which has been shown to stimulate chondrocyte proliferation e.g. in rabbit articular chondrocytes [70], a mechanism has recently been proposed for rat chondrocytes [71]. This could be one aspect why TGF-β alone is not suited to induce cartilage formation. BMP-6, on the contrary, has been demonstrated to reduce proliferation by induction of differentiation [72], a process opposed to cell growth in articular cartilage. The overall increased growth may be indicative of a still to large influence of TGF- β, the balance between the two factors still has to be adjusted for successful hyaline cartilage formation.

A strong indication for the chitosan’s capacity to support the induction of chondrogenesis by growth factors was the detection of both glycosaminoglycan (GAG) synthesis, a part of the extracellular matrix of cartilage, as well as collagen type II transcription and translation together with *ACAN* and *SOX9* upregulation. Human ADSCs cultured on chitosan scaffolds with normal medium did not show substantial formation of GAGs compared to both those cultured in standard or modified chondrogenic medium and the corresponding 3D pellet controls. This was in accordance with results of immunofluorescent, immunohistochemical, histomorphometrical analysis and qRT-PCR assays. *COL2A1* was significantly down-regulated by day 28 in the NS group with *ACAN* and *SOX9* also decreasing substantially compared to the same normal medium group with 3D-pelleted hADSCs. The relative gene expression assay of chondrogenic differentiation markers further supported this finding where *ACAN* and *SOX9* expression increased in both chitosan groups irrelevant of medium type, even though *COL2A1* was only significantly upregulated in chondrogenic medium. The transcription factor *SOX9* is an early marker for chondrogenesis that regulates collagen type II and cartilage-specific matrix synthesis by activating the *COL2A1* and *ACAN* [73, 74]. Another study previously demonstrated that *SOX9* was also expressed in proliferating and pre-hypertrophic chondrocytes, but is downregulated in hypertrophic chondrocytes [75]. From the present results, it appears that the chitosan scaffold on its own can induce hADSCs to undergo differentiation towards a cartilage lineage but cannot form a cartilage matrix without the addition of a subsequent stimulant, which was the case for the CS, CS + hBMP-6 and CP groups where hTGF-β_3_ alone or in combination with hBMP-6 was used. Indeed, through the addition of hBMP-6, *SOX9* significantly increased and was greater than the standard CS group, a pattern that remained consistent for all gene types including *ACAN* and *COL2A1*.

Immunofluorescence staining of collagen type II and aggrecan as well as histological staining with Alcian blue for GAG confirmed that the groups treated with hTGF-β_3_, CP and CS and hTGF-β_3_ + hBMP-6 showed an increase both on the translational and transcriptional levels of most cartilage relevant markers. In particular in the chitosan group, histological and immunofluorescence staining demonstrated that GAG and collagen type II synthesis was significantly increased during the in vitro culture over 28 days, indicating that indeed this biomaterial supports the formation of specific matrices in this case those consisting of collagen type II as is the case for cartilage. *ACAN*, *COL2A1* and *SOX9* expression levels all increased significantly, suggesting that the chitosan scaffold alone provides a far superior microenvironment that allows for the adhesion, proliferation and differentiation of cells under the influence of both chondrogenic medium types, especially with a combination of hBMP-6 and hTGF-β_3_.

However, one crucial question in cartilage regeneration in vitro is whether the matrix formed is hyaline or contains significant amounts of collagen I, and if it is hyaline matrix does it develop towards stable articular cartilage or rather progresses towards hypertrophy and mineralization. *ACAN*, *COL2A1* and *SOX9* are markers that are generally utilized to monitor if any cartilage formation had occurred irrelevant of the type [76–78]. *COL1A1*, *COL10A1* and *COMP* on the other hand are classical markers to further differentiate what type of cartilage is being formed [77, 79, 80]. Articular cartilage has superior load-bearing and mechanical properties and is free of collagen I [81, 82], while hyaline cartilage formed during endochondral ossification of embryogenesis for certain skeletal bones, is characterized by the early appearance of collagen X [83]. In our study, collagen type I immunofluorescence/histochemical staining was minimally detected in NS and CS but not CS + hBMP-6 at day 7, 14 and 28, strengthening our previous articular hyaline cartilage formation discoveries [31]. This corroborated with the gene expression patterns for *COL1A1* suggesting that cultures were not purely articular and already programmed towards hypertrophy, except for the chitosan scaffolds cultured in the modified hTGF-β_3_ + hBMP-6, where these genes were all significantly and consistently down-regulated (Fig. 9b, d). Whilst *COL10A1* was downregulated with immunohistochemical staining validating gene expression results, *COL1A1* decreased significantly in the standard chondrogenic chitosan or whilst in the pellet culture. *COL1A1* remained high and only after day 28 had minimally decreased, but not significantly, as is expected during articular cartilage formation. Whilst stem cells, in the absence of a carrier matrix, could have a higher probability to become articular chondrocytes in the presence of hTGF-β_3_, the latter’s cartilage formation potential is far too low to be considered on its own for large scale in vivo cartilage repair. It appeared that the chitosan scaffolds creates a superior environment to support large scale cartilage matrix production, provided that the correct morphogens are present to facilitate the correct matrix formation [13, 14]. This was made evident by the expression of COMP, a pentameric non-collagenous matrix protein expressed primarily in articular cartilage. It is reported to regulate both chondrogenesis and endochondral ossification, including stabilizing the ECM of articular cartilage, by maintaining the structural integrity through its interaction with ACAN, COL2A1, COL10A1 and fibronectin [84–86]. Results shown here in part recapitulate our previous observations [31], with *COMP* being downregulated as compared to unstimulated hADSCs in the monolayer in all groups except for the CS and CS + hBMP-6 groups cultured for 7 (CS + hBMP-6 and CS) and 14 (CS only) days. During the first 7 days, *COMP* expression was not different between CS and CS + hBMP-6. Only by Day 14, *COMP* in the CS + hBMP-6 was downregulated here with the CS group only following this trend by day 28. This suggests that TGF-β_3_ on its own enhances the production of an extracellular matrix that is however not articular in nature as evidence by the expression of *COL1A1* and *COL10A1*. At Day 28, *COMP* group decreased in its expression pattern in both CS and CS + hBMP-6 groups, similar to observations reported before [87]. We suggest that the balance between the TGF- β_3_ and BMP-6 was not adjusted well enough to avoid the decrease in the medium containing both growth-factors. COMP is a matrix element with several important functions, among which there is an important role in adipose tissue [88], the source of the stem cells used. Possibly, depending on the donor, cells had maintained a high *COMP* expression and the observed downregulation was indeed a regulation to normal levels.

Indeed, the results further demonstrated clearly that hTGF-β_3_ alone is not a suitable morphogen for articular cartilage formation as has been suggested before but ignored for most part in the tissue engineering field [89–91]. Our results unequivocally demonstrated that hTGF-β_3_ alone causes endochondral cartilage formation tending towards ossification rather than true articular cartilage development, unless it is counteracted or synergizes with another TGF-β supergene family member(s) that modulate better the process of articular cartilage formation, here hBMP-6. Although the current literature does not explicitly state this, as it generalizes more on the term chondrogenesis [47, 48], we can now with a better degree of certainty claim that the synergetic effect of hBMP-6 with hTGF-β_3_ causes “articular” chondrogenesis.

The TGF-β isoforms have previously been established to supposedly possess good “chondrogenic differentiation potentials” when utilizing MSCs [66] as much of the TGF-β isoforms are present in articular cartilage with even miniscule quantities of active TGF-β being a potent stimulant for proteoglycan and type II collagen synthesis. This was again substantiated in the present study where 10 ng/ml of hTGF-β_3_ promoted significant chondrocyte differentiation and resulted in increased type 2 collagen transcription and translation [16] but in hADSCs. Indeed, most of the previous studies on the articular cartilage differentiation potential were based on MSCs, and were solely focused on whether TGF-β isoforms also possess similar chondrogenic differentiation capacitates as ADSCs [92]. Whilst in vitro traits of TGF-β isoforms at inducing articular chondrogenesis show increased *COL2A1*, *SOX9*, *ACAN* and reduced *COL1A1* expressions [93, 94], in vivo based research remains problematic; despite extensive investigations demonstrating the potential of MSCs to regenerate cartilage, the latter degenerates quickly in vivo after a certain number of weeks, leading to ossification rather than maintaining articular cartilage state [60, 94]. The question must therefore be asked whether the matrix was articular to begin with. Indeed, in the present study similar gene expression results with respect to *COL2A1*, *SOX9* and *ACAN* were produced in hADSCs too, supporting the concept that TGF-β isoforms, especially hTGF-β_3_, can induce a form of chondrogenic differentiation also in these stem cells [92]. However, the exception of *COL1A1* at day 28 in both pelleted hADSCs in the absence of standard chondrogenic medium (Fig. 9b) and the enhanced expression of *COL1A1* and *COL10A1* in the biomimetic chitosan scaffolds, replicating in part an in vivo environment, would suggest that previous theories are not quite as accurate, possibly due to outdated qPCR techniques [65, 69], or that not all stem cells share equal cellular differentiation capabilities to undergo transformation into certain cell types for the formation of specialized tissue matrices. Even in studies using certain BMP members such as BMP-7, BMP-2 and BMP-4 alone as part of chondrogenic medium instead of a TGF-β isoform, promising in vitro results were achieved [95–98], but for in vivo applications the newly formed cartilage structures revert to the typical ossification of endochondral bone formation [99]. This suggests that single morphogen applications are not sufficient at achieving the correct cellular response to yield correct tissue formation especially where articular cartilage formation is concerned. Only by combining two morphogens of the TGF-β supergene family members, here hTGF-β_3_ with hBMP-6, can proper articular cartilage formation be re-established to produce stable hyaline cartilage, as shown in the present study and previously postulated by [43] and us [31, 69, 100].

It therefore appears that, despite the results proving that chitosan is a suitable carrier with a potential to support articular cartilage formation, the appropriate morphogens are the key driving force for the induction of correct matrix formation. In this model, even in the presence of a suitable carrier matrix, the correct “keys” are needed to open the locking mechanism of correct tissue morphogenesis. Our findings confirm that hTGF-β_3_ alone is not suited as an articular cartilage-inducing morphogen for hADSCs. Previous concepts may need to be re-evaluated in light of modern molecular techniques based on advanced qRT-PCR techniques as stated by Bustin et al. [101] or Next Generation Sequencing that will provide detailed molecular mechanistic insights into the underlying mechanisms of single TGF-β supergene family members and multiple combined morphogen members. Such efforts will help properly identify how these factors act on causing stem cell differentiation towards articular chondrocytes. Future research on the regulation of these proteins and the identification of intrinsic cellular pathways leading to proper articular cartilage formation are therefore critical to avoid errors or misinterpretations that lead to further delays in clinical applications. Future articular cartilage regeneration procedures should consider including hTGF-β_3_ + hBMP-6, and find ways to maintain the presence of these morphogens for at least 28 days or more. This, if achieved, would very likely lead to articular cartilage matrix formation that does not degenerate in the future to fibrocartilage or osteogenic tissue.

## Conclusion

Chitosan remains a viable and highly beneficial biomaterial that has the capacity to support extensive differentiation of stem cells into chondrocytes, but may tend towards ossification unless the correct signals are present. Human ADSCs have been shown to be a competitive alternative stem cell type that has many excellent qualities to differentiate into the appropriate cell types including chondrocytes, provided that the correct signaling molecules are present. Human TGF-β_3_, however, does not properly facilitate articular cartilage chondrocyte differentiation in hADSCs in pellet or on a biomaterial, rather tending towards inducing endochondral cartilage formation that will in the long run ossify. Moreover, our results confirmed that only by using an optimized morphogen mixture together with a suited carrier matrix may provide a more efficient environment at regenerating articular cartilage defects than existing strategies, as hBMP-6 with hTGF-β_3_ maintains an articular cartilage formation milieu.

## Methods

### Biomaterial scaffold design

Porous sponges were manufactured by lyophilization of glutaraldehyde-cross-linked 0,5% wt/vol chitosan hydrogels as described previously [31]. Briefly, chitosan with a 95% degree of deacetylation (Heppe Medical, Halle, Germany) was dissolved at 1% wt/vol in 0.1 N HCl with pH 1. Using 1 N NaOH, the pH was carefully adjusted to 5 under constant stirring and dropwise addition of the base. Hydrogels were formed by mixing 1 ml of chitosan solution with 1 ml aqueous 1% glutaraldehyde solution (Sigma-Aldrich, St. Louis, MI, USA) in hollow disc shapes with a diameter of 15 mm. After gelation, samples were frozen at − 32 °C using polystyrene insulation to control freezing rate. Frozen samples were then freeze-dried at − 50 °C under vacuum using an Alpha 1–4 LD system (Christ, Osterode am Harz, Germany). The dry scaffolds were then trimmed on both ends to a final height of 8 mm with a microtomic blade and gamma-sterilized at ca. 27 kGy.

The resulting scaffold morphologies were examined using a VHX-5000 3D digital microscope (Keyence, Osaka, Japan) and software VHX-5000 Ver. 1.6.1.0 / System Ver. 1.04 (Keyence). The microstructure of the scaffolds was observed by scanning electron microscopy (SEM) (JEOL JSM-6360LV, Tokyo, Japan).

### Isolation and culture of hADSCs

Human ADSCs were isolated, as previously described [31, 102], from subcutaneous adipose tissue from 4 patients of different sex and age (age 31–80, BMI 29–52) that was acquired from the Biobank of the University Hospital of Munich Germany which operates in accordance to the European Union compliant ethical and legal framework of the Human Tissue and Cell Research Foundation (http://www.htcr.org). The research was approved by the Human Ethics Committee of the Faculty of Medicine at the University of Munich and the Bavarian State Medical Association. Briefly, harvested adipose tissue were rinsed with phosphate buffered saline (PBS) containing 180 IU/ml penicillin/streptomycin and 0.75 μg/ml amphotericin B (Biochrom, Berlin, Germany), after which the tissue was cut into small fragments and digested with 0.2% collagenase A solution (Sigma-Aldrich) in DMEM (Gibco, Waltham, MA, USA) at 37 °C. Then 15% fetal calf serum (FCS; Sigma-Aldrich) supplemented culture medium was added, after which the mixture was resuspended, filtered through 100 μm sieves and centrifuged at 400 g for 10 min at room temperature (RT). The pellet containing hADSCs was resuspended with fresh growth medium (DMEM, 15% FCS, 60 IU/ml penicillin/streptomycin), seeded in a T-75 culture flask and cultured at 37 °C with 5% CO_2_ for 24 h. Subsequently, the adhered cells were washed with PBS and 20 ml of fresh growth medium was added. The medium was replaced every 3 days. Human ADSCs used in this study were used at passage 4.

### Cell seeding onto chitosan sponges and in vitro chondrogenic differentiation

The dry scaffolds were placed carefully in a 12-well plate (Thermo fisher scientific, Waltham, MA, USA) and covered with 2 ml normal growth medium (high-glucose DMEM 4.5 g/L D-glucose, 110 μg/ml Pyruvate; Gibco, supplemented with 10% FCS, 60 IU/ml penicillin/streptomycin). Scaffolds were then incubated at 37 °C with 5% CO_2_ for 6 h after which the medium was changed and left to incubate overnight. Human ADSCs (~ 90% confluent) were digested with trypsin/EDTA and counted and resuspended at a concentration of ~ 1 × 10^7^/ml. To seed the cells on the chitosan scaffolds, the old medium was removed and 100 μl of cell suspension was pipetted evenly onto each. Scaffolds with cells were incubated at 37 °C at 5% CO_2_ for 1 h to allow for cell attachment, whereupon 2 ml of normal growth medium was added to each well and incubated overnight. The following morning (Day1), the cell-seeded scaffolds were transferred into either normal growth medium (Normal + Scaffold or “NS”; *n* = 9), standard chondrogenic (hTGF-β_3_) medium (Chondrogenic + Scaffold or “CS”; *n* = 9) or modified chondrogenic (hTGF-β_3_ + hBMP-6) medium (Chondrogenic + hBMP-6 + Scaffold or “CS + hBMP-6”; *n* = 9). The standard chondrogenic medium was normal growth medium supplemented with 10 ng/ml recombinant human TGF-β_3_ (R&D Systems, Minneapolis, MN, USA), 100 nM dexamethasone (Sigma-Aldrich), 50μg/ml L-ascorbic acid-2-phosphate (Sigma-Aldrich), 40μg/ml L-proline (Sigma-Aldrich) and ITS+ 1 (Sigma-Aldrich; final concentrations: 10 mg/L insulin, 5.5 mg/L transferrin, 4.7 μg/ml linoleic acid, 0.5 mg/ml bovine serum albumin and 5 μg/L selenium). Chondrogenic + hBMP-6 medium was the standard chondrogenic medium as described above + 10 ng/ml recombinant hBMP-6 [22]. The cell-seeded constructs cultured with normal growth medium (NS) were considered as scaffold control group. Samples were cultured for 7, 14, and 28 days and medium was replaced every 3 days.

### Pellet culture and chondrogenic differentiation

Pellet culture was used as comparable, scaffold-free 3D culture control [103], to investigate both scaffold and culture medium influence on stem cell differentiation and matrix formation. Human ADSCs from the fourth passage were resuspended at a concentration of 2.5 × 10^5^ cells per ml in normal growth medium (see above). Two milliliters of the cell suspension containing 5 × 10^5^ hADSCs were transferred into a 15 mL polypropylene conical tube and centrifuged at 500 x g for 5 min to allow for 3D cell pellet formation. The 3D pelleted cells were then incubated overnight at 37 °C with 5% CO_2_ with loosened caps to permit gas exchange. Spheroid aggregates formed at the bottom of each tube. Next day (Day1), the culture medium was replaced with 2 ml of fresh growth medium (Normal + Pellet or “NP”; n = 9), standard chondrogenic (hTGF-β_3_) medium (Chondrogenic + Pellet or “CP”; n = 9) or modified chondrogenic (hTGF-β_3_ + hBMP-6) medium (Chondrogenic + hBMP-6 + Scaffold or “CS + hBMP-6”; n = 9) carefully so as not to resuspend the cell pellet. The 3D pellet medium was changed every 3 days, and 3D cell pellets were cultured for 7, 14, and 28 days prior to harvest and processing for analysis. Cells cultured in normal growth medium were used as the pellet control group.

### Scanning electron microscopy (SEM)

Scanning electron microscopy was performed as previously described [31]. In order to see the matrix development progression of hADSCs on the scaffolds treated with modified chondrogenic medium, a scaffold was randomly chosen and cultured for 1, 7, 14 and 28 days. Upon harvest, the cell-scaffold constructs were washed with PBS and fixed in 2.5% glutaraldehyde in PBS overnight at 4 °C. The constructs were then stained with 1% osmium tetroxide, dehydrated in a graded series of alcohols, dehydrated using the critical point drying method, and coated with gold. The samples were examined with a scanning electron microscope (SEM) at an accelerating voltage of 20 kV (Carl Zeiss EVO LS 10, Oberkochen, Germany).

### Cell viability and proliferation assay

The viability and proliferation of hADSCs cultured in the CHI and CHI/HA scaffolds were evaluated by means of a water-soluble tetrazolium-1(WST-1) reagent (Roche, Basel, Swiss) in combination with Quant-iTTM PicoGreen dsDNA Kit (Invitrogen, California, USA) at day 1 and subsequently at day 7, 14, or 28, as described previously [31]. Briefly, the CHI and CHI/HA scaffolds with the hADSCs were transferred to a new 24-well plate and washed twice with PBS after which 0.5 ml fresh normal growth medium containing WST-1 at 10:1 (v/v) was added to each well and incubated for 3 h at 37 °C at 5% CO_2_. The absorbance of the WST-1/medium mixture was read at 450 nm using a Synergy HT microplate reader and Gen 5 2.03 software (BioTek, Vermont, USA) in a 96-well plate. The same scaffolds were used for the PicoGreen dsDNA Assay. Here The 1% PSCs were washed twice with PBS. According to the manufacturer’s protocol, the cells were lysed from the scaffold and DNA standards were mixed with TE-buffer and subsequently with Quant-iT PicoGreen dsDNA reagent. The samples were excited at 480 nm and the fluorescence emission intensity was measured at 520 nm using a Synergy HT microplate reader and Gen 5 2.03 software (BioTek).

### Cell survival in the scaffold

The effects of chitosan scaffolds on cell survival in both normal and altered chondrogenic differentiation medium including normal growth medium were studied by using a Live/Dead assay, as described previously [31]. At day 1, 7, 14 and 28, the cell-scaffold constructs were stained with a LIVE/DEAD Viability/Cytotoxicity Kit (Invitrogen,). Briefly, cell-scaffold constructs were rinsed with PBS and incubated in staining solution containing Calcein AM and Ethidium homodimer-1(EthD-1) at room temperature for 30 min, followed by washing with PBS. Then the constructs were examined by fluorescence microscopy (Carl Zeiss). Healthy cells fluoresce green, while the nucleus of dead cells fluoresced red [89].

### Histological, immune-fluorescence and -histochemical analysis

As described previously [31], after 7, 14 and 28 days of culture the cell-scaffolds and 28 day 3D cell pellets were fixed in 4% paraformaldehyde for 30 min at room temperature. The 1% chitosan scaffolds with cells were dehydrated through a graded series of alcohols into paraffin, whereas the 3D cell pellet cultures were embedded in a Tissue-Tek O.C.T.™ compound (Sakura Finetek Germany, Staufen, Germany) and frozen in liquid nitrogen. Following this, 10 μm thick sections were cut using either a Microtome (Leica, Wetzlar, Germany), for paraffin specimens, or a CM 3050 cryomicrotome (Leica), for the cryogenic embedded specimens. To visualize tissue morphology and cartilage matrix formation, sections were stained with Alcian blue at pH 2.5 for glycosaminoglycan (GAG) content formation. All stained sections were analyzed with a PreciPoint M8 Digital Microscope & Scanner (PreciPoint GmbH, Freising, Germany).

To determine the quality of the matrix composition, chitosan scaffold cultures within the NS, CS and CS + hBMP-6 groups were immunofluorescenctly stained for collagen I, collagen II, aggrecan and immunohistochemically for collagen I and collagen X (for validation purposes). Briefly, paraffin sections were incubated with primary antibodies (all from Abcam, Cambridge, UK) for either collagen type I (1:300; Cat# ab34710), collagen type II (1:200; Cat# ab34712), aggrecan (1:300; Cat# ab3778) and collagen type X (1: 200; Cat# ab58632) at 4 °C overnight. The antibodies were diluted with antibody dilution buffer (DCS, Germany). For negative controls, the first antibody was omitted. The slides were then incubated with the conjugated secondary antibody (Abcam, Cambridge, UK) for 1 h at room temperature. For immunofluorescence nuclei of cells were then stained for 8 min with Hoechst 33342 (Life Technologies, Carlsbad, USA). The slides for immunofluorsence were mounted with Fluoromount W (Serva Electrophoresis, Heidelberg, Germany) air-dried and stored in darkness at 4 °C, whereas those for immunohistochemical assessment where mounted with chromogen AEC (DCS, Germany). Fluorescence microscopy was then performed with a Zeiss Axioskop 40 equipped with appropriate filter sets and AxioCam MRc 5 (Carl Zeiss, Munich, Germany). Images were obtained with Axio Vision, Rel. 4.9 (Carl Zeiss, Munich, Germany). Exposure time was kept constant for the samples where fluorescence intensity was to be compared. On the other hand, immunohistochemical stained sections where captured using PreciPoint M8 Digital Microscope & Scanner (PreciPoint) with AEC stained tissue staining as a wine-reddish color.

### Histomorphometrical assessment Alcian blue GAG content

Histological cuts were processed for Alcian blue positive matrix formation using a combination of image processing codes based on ImageJ (https://imagej.nih.gov/ij/). The pictures were first treated for noise using a Gaussian filter (kernel = 2.0, sigma = 0.5), and then segmented with a color threshold in the Red-Green-Blue color space, selected visually to isolate pixels stained with Alcian blue within the histological pictures and setting them to 1, while other region were set to 0.. Image parts not within the specimen region were removed. The final %GAG content was calculated as the ratio of pixels within the Alcian blue positive region to the total amount of pixels within the image, after subtraction of the external region. A mean and standard deviation was calculated for each group at each time point, and a Student’s T-Test was performed for statistical differences between groups and at each time point (significance if *p* < 0.05).

### Quantitative real-time PCR (qRT-PCR)

As described previously [31], quantitative RT-PCR, according to the MIQE guidelines [101], was performed to determine the relative expression of the chondrogenic genes, *aggrecan* (*ACAN*), *collagen type II* (*COL2A1*), *cartilage oligomeric matrix protein* (*COMP*), *SRY-box 9* (*SOX9*) with *collagen type I* (*COL1A1*) and *collagen type X* (*COL10A1*) being incorporated to determine if cartilage matrix development was pure articular or progressing towards an endochondral ossification lineage. After 7, 14 and 28 days, total RNA was isolated using a modified RNA Trizol extraction procedure [104]. Briefly, 1 ml Trizol (Invitrogen) was added to cell material after which chloroform (Sigma-Aldrich) was added to permit separation of the RNA from the proteinaceous material. After centrifugation the aqueous RNA containing phase was transferred to a fresh tube where the RNA was then precipitated out by adding Isopropanol (Sigma-Aldrich). After incubation at RT for 10 min samples were centrifuged at 16000 rpm over night at 4 °C, upon which RNA pellets were then washed with 75% Ethanol (Merck, Billerica MA, USA) and permitted to dry briefly to prevent alcohol contamination. After drying, total RNA was resuspended in 32 μl RNase free water (Gibco) after which the concentration and purity of the RNA was determined using a NanoDropTMLite spectrophotometer (Thermo Scientific) and quality assessed with a Bioanalyzer 2100 (Agilent Technologies). After RNA extraction approximately 1 μg of RNA was reverse transcribed into complementary DNA (cDNA) utilizing the QuantiTect Reverse Transcription cDNA Synthesis Kit (Qiagen, Germany).

Quantitative RT-PCR was then performed in duplicate, using the FastStart Essential DNA Green Master (Roche) on a Light Cycler 96 thermocycler (Roche). Each reaction mixture contained: 10 ng cDNA, 10 μM of each primer (Table 1), 2x FastStart Essential DNA Green Master and RNase-free water to a final reaction volume of 20 μl. The primers of six target genes were designed using Gene fisher v. 2.0 (http://bibiserv.techfak.uni-bielefeld.de/genefisher2) and optimized according to the MIQE Guidelines [101]. Use of GeNorm (http://medgen.ugent.be/~jvdesomp/genorm/) established that *TATA sequence binding protein* (*TBP*), *succinate dehydrogenase complex flavoprotein subunit A* (*SDHA*), *ribosomal protein lateral stalk subunit P0* (RPLP0) and *Ribosomal Protein L13a* (*RPL13a*) were the most appropriate internal reference genes to use in this experiment. All amplified PCR products underwent sequencing (GATC Biotech, Constance, Germany) to confirm that the correct sequence had been amplified. Quantitative RT- PCR cycling conditions included a 3 min pre-incubation at 95 °C, followed by a three step amplification program of 40 cycles consisting of a denaturation, annealing and extension step set at 95 °C for 10 s, 58 °C for 15 s and 72 °C for 30 s, respectively. Six target genes relative expression between samples was normalized to the four reference genes using the qbase+ software (https://www.qbaseplus.com). Data was further normalized to untreated hADSCs in monolayer.

**Table 1:** Gene specific primers used for quantitative real-time PCR

Gene	Forward primer (5′- 3′)	Reverese primer (3′ - 5′)	Accession Nr.	Amplicon Size (bp)
*COL2A1*	GCCCAGTTGGGAGTAAGT	CACCAGGATTGCCTTGAA	NM_001844.4	106
*COL1A1*	GCTGGTCCTCCAGGTGAA	GGGGACCAACAGGACCA	NM_000088.3	159
*COL10A1*	TGGCCTGCCTGACTTTA	AATGTCCAGCTCACTGGA	NM_000493.3	151
*ACAN*	ACCCAAGGACTGGAATCT	CCTGATCCAGGTAGCCTT	NM_001135.3	149
*COMP*	TGCACCGACGTCAACGA	CCGGGTGTTGATGCACA	NM_000095.2	231
*SOX9*	GTGGCTGTAGTAGGAGCT	GCGAACGCACATCAAGA	NM_000346.3	155
*ACTB* (reference)	CTGCCCTGAGGCACTC	GTGCCAGGGCAGTGAT	NM_001101.3	197
*RPLP0* (reference)	CAACCCAGCTCTGGAGA	CAGCTGGCACCTTATTGG	BC001834.2	116
*TBP* (reference)	CACTTCGTGCCCGAAAC	GCCAGTCTGGACTGTTCT	BC110341.1	121
*POLR2e* (reference)	CTATCTGGTGACCCAGGA	CTGCAGAAACTGCTCCA	J04965.1	322

### Statistics

As described previously [31], data is presented as means ± standard deviation (SD, *n* = 9) for the results of WST-1, PicoGreen and qPCR. Qbase+ software was used to analyze the data from qPCR. Microsoft Excel and Prism 5.02 software (GraphPad Software, San Diego, USA) were used for analyzing the data. An ANOVA and a Kruskal-Wallis test were performed to test for the overall effect of the variance using R software version 3.6.1 (SAS, Marlow, UK). The Students T-test was used post hoc for comparing groups of data. A *p* < 0.05, *p* < 0.01 and *p* < 0.001 were considered significant, highly or extremely significant, respectively. Statistical significance was indicated by * for p < 0.05, **for p < 0.01 and ***for *p* < 0.001.

## Supplementary information

**Additional file 1: Supplementary Figure 1.** Immunohistochemical staining of collagen type I (black arrows, wine red color) at day 7, 14 and 28 in chitosan scaffolds with hADSCs cultured in normal (NS), standard chondrogenic (CS) or modified chondrogenic + hBMP-6 medium (CS + hBMP-6). The chitosan scaffolds are a brownish colour, whereas living cell nuclei and matrix are a pinkish. Magnification set a 10x.

**Additional file 2: Supplementary Figure 2.** Immunohistochemical staining of collagen type X at day 7, 14 and 28 in chitosan scaffolds with hADSCs cultured in normal (NS), standard chondrogenic (CS) or modified chondrogenic + hBMP-6 medium (CS + hBMP-6). The chitosan scaffolds are a brownish colour, whereas living cell nuclei and matrix are a pinkish. Magnification set a 10x.

**Additional file 3: Supplementary Figure 3.** Relative gene expression quantity of (A) ACAN, (B) COL1A1, (C) COL2A1, (D) COL10A1, (E) COMP and (F) SOX9 between all-time points (day 7, 14 and 28) per 3D pellet or chitosan scaffolds culture medium group (N = normal medium; C = chondrogenic medium, *P* = 3D Pellet; S = chitosan scaffolds,). (*p < 0.05, **p < 0.01, ***p < 0.001). The baseline 0 represents untreated hADSCs in monolayer, which was the normalisation factor.

## Data Availability

The necessary algorithmic codes of the program GeNorm are readily available at (https://www.researchgate.net/publication/343548360_GeNorm_v35_xl; https://genorm.cmgg.be/). All data, raw and processed, is readily available from the corresponding author on request.

## Abbreviations

3D: Three-Dimensional
ECM: Extracellular Matrix
MSC: Mesenchymal stem cells
hADSC: Human adipose-derived stem cells
h: Hour
min: Minutes
GAG: Glycosaminoglycan
DMEM: Dulbecco’s modified eagle medium
SEM: Scanning electron microscope
qRT-PCR: Quantitative real-time polymerase chain reaction
hTGF-β_3_: Human transforming growth factor beta 3
hBMP-6: Human bone morphogenetic protein 6
WST-1: Water soluble tetrazolium - 1
NCHI: Chitosan in normal growth medium
NP: Pellet in normal growth medium
CP: Pellet in standard chondrogenic growth medium
ACAN: Aggrecan
COL1A1: Collagen type I
COL2A1: Collagen type II
COL10A1: Collagen type X
SOX9: SRY-box 9
COMP: Cartilage oligomeric matrix protein
NRQ: Normalized relative quantity
TBP: TATA sequence binding protein
SDHA: Succinate dehydrogenase complex flavoprotein subunit A
RPLP0: Ribosomal protein lateral stalk subunit P0
RPL13a: Ribosomal Protein L13a
PBS: Phosphate buffered saline
FCS: Fetal calf/bovine serum
SD: Standard deviation
CS: Chitosan scaffold in standard chondrogenic growth medium
NS: Scaffold in normal growth medium
