# Supplementary figures and images for: Synergistic interaction of hTGF-β3 with hBMP-6 promotes articular cartilage formation in chitosan scaffolds with hADSCs: implications for regenerative medicine
Source: BMC Biotechnol. 2020 Aug 27;20:48. doi: 10.1186/s12896-020-00641-y (PMC7457281; doi:10.1186/s12896-020-00641-y)

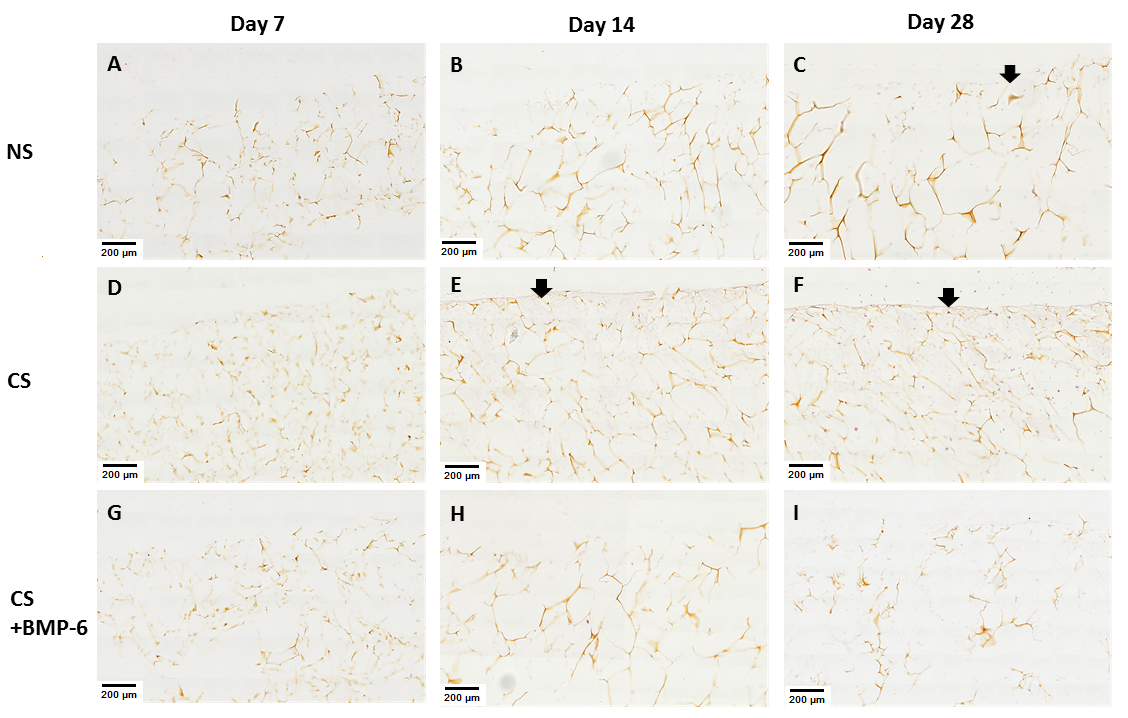

Supplement: Supplementary file 1 — Additional file 1: Supplementary Figure 1. Immunohistochemical staining of collagen type I (black arrows, wine red color) at day 7, 14 and 28 in chitosan scaffolds with hADSCs cultured in normal (NS), standard chondrogenic (CS) or modified chondrogenic + hBMP-6 medium (CS + hBMP-6). The chitosan scaffolds are a brownish colour, whereas living cell nuclei and matrix are a pinkish. Magnification set a 10x. [file 12896_2020_641_MOESM1_ESM.tif]

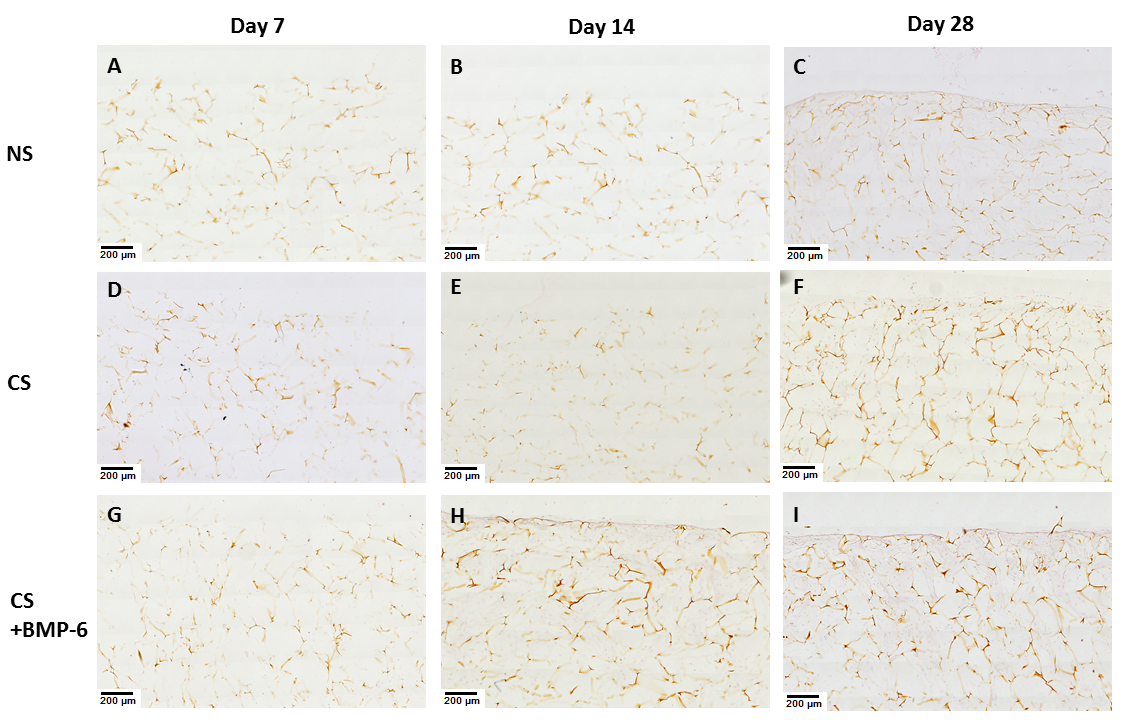

Supplement: Supplementary file 2 — Additional file 2: Supplementary Figure 2. Immunohistochemical staining of collagen type X at day 7, 14 and 28 in chitosan scaffolds with hADSCs cultured in normal (NS), standard chondrogenic (CS) or modified chondrogenic + hBMP-6 medium (CS + hBMP-6). The chitosan scaffolds are a brownish colour, whereas living cell nuclei and matrix are a pinkish. Magnification set a 10x. [file 12896_2020_641_MOESM2_ESM.tif]
